# Supplementary material for: Pretreatment tumor-related leukocytosis misleads positron emission tomography-computed tomography during lymph node staging in gynecological malignancies
Source: Nat Commun. 2020 Mar 13;11:1364. doi: 10.1038/s41467-020-15186-z (PMC7069975; doi:10.1038/s41467-020-15186-z)
Supplement: Supplementary file 1 — Supplementary Information [file 41467_2020_15186_MOESM1_ESM.pdf]

**Supplementary Information for**

# Pretreatment tumor-related leukocytosis misleads positron emission tomography-computed tomography during lymph node staging in gynecological malignancies

Mabuchi et al.

Supplementary Fig. 1

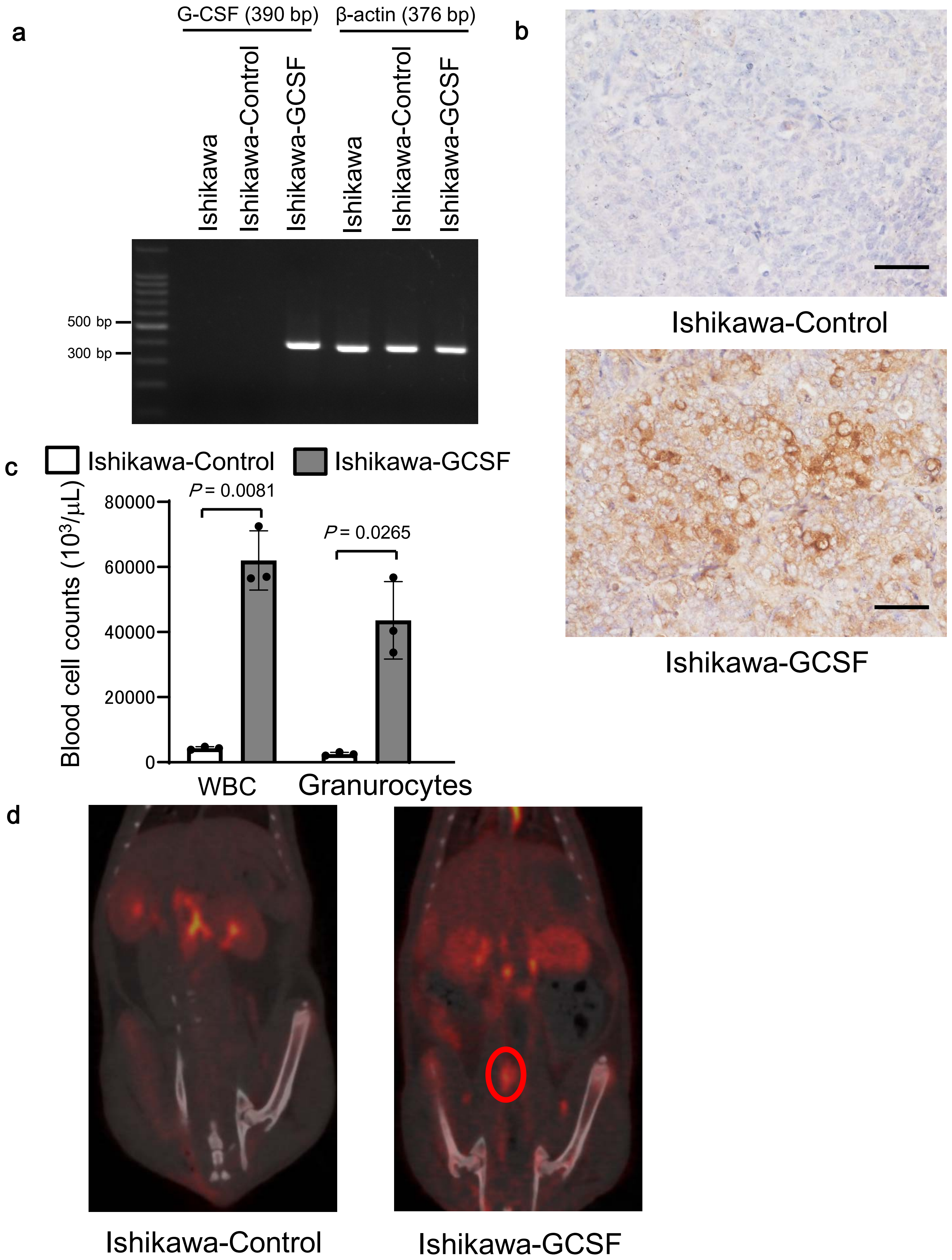

e

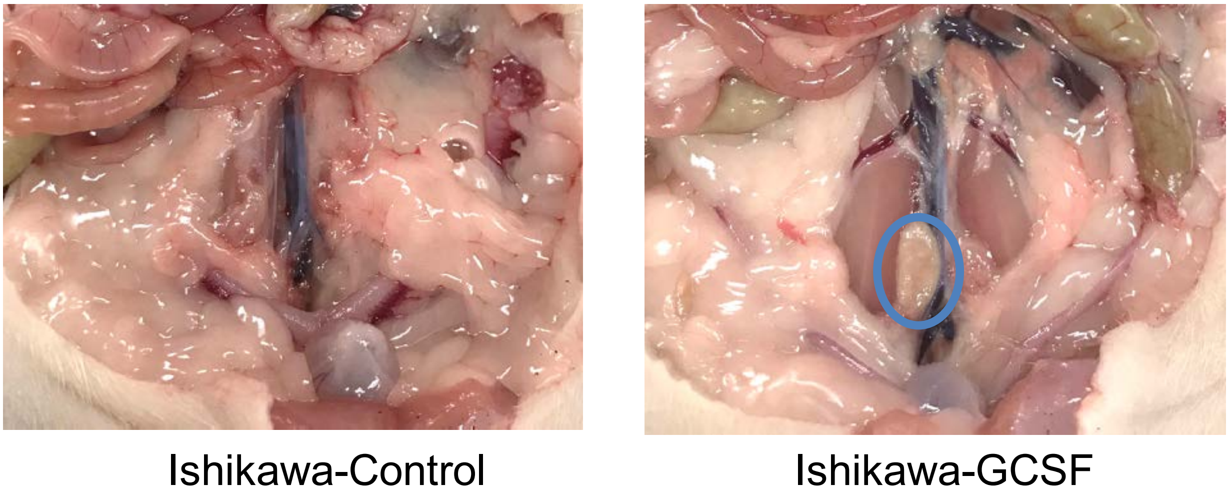

Ishikawa-Control
Ishikawa-GCSF


f

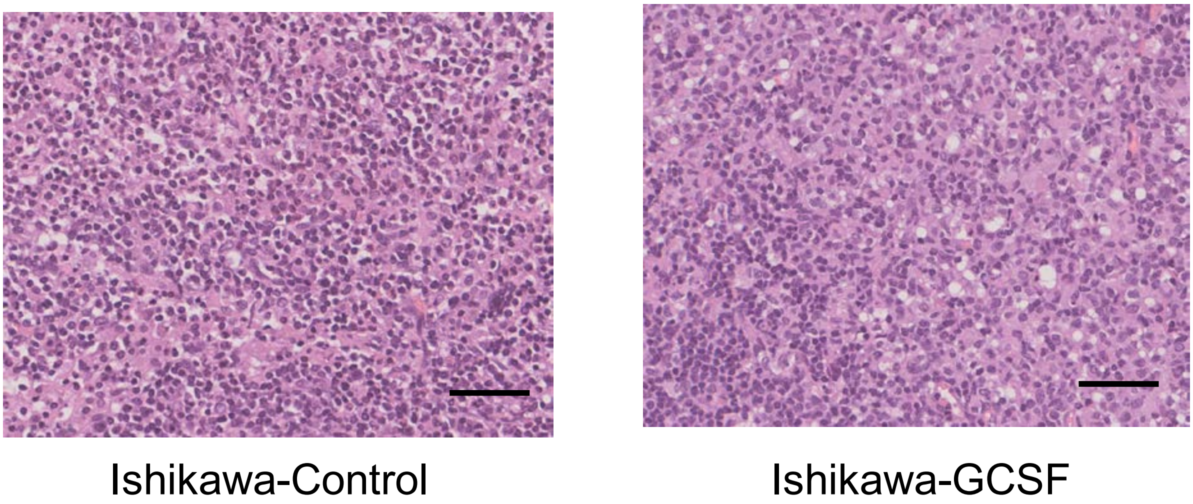

Ishikawa-Control
Ishikawa-GCSF


g

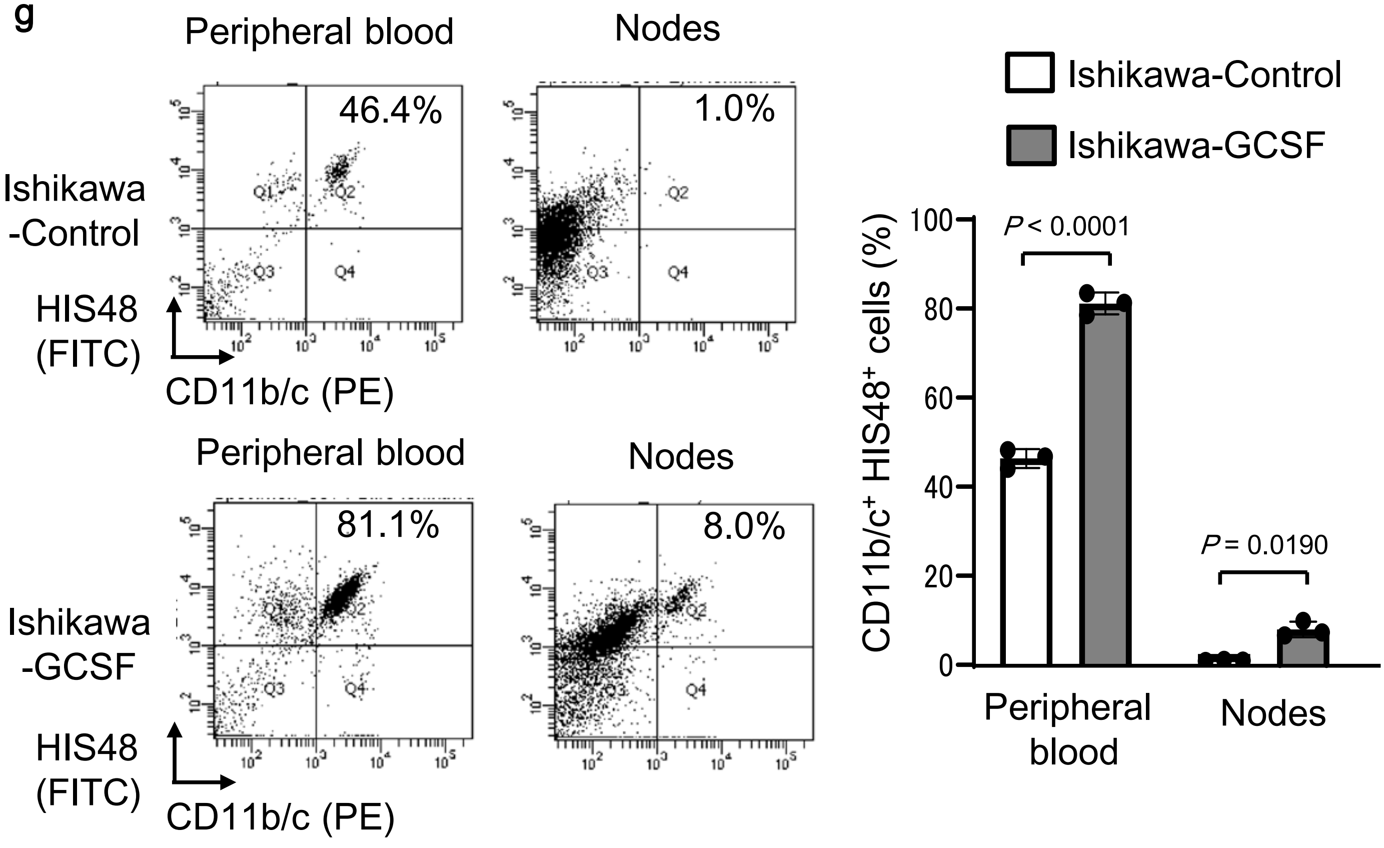

Peripheral blood
Nodes
Ishikawa -Control
46.4%
1.0%
Q1
Q2
Q3
Q4
HIS48 (FITC)
CD11b/c (PE)
Ishikawa -GCSF
81.1%
8.0%
Ishikawa-Control
Ishikawa-GCSF
CD11b/c+ HIS48+ cells (%)
100
80
60
40
20
0
P < 0.0001
P = 0.0190
Peripheral blood
Nodes

h

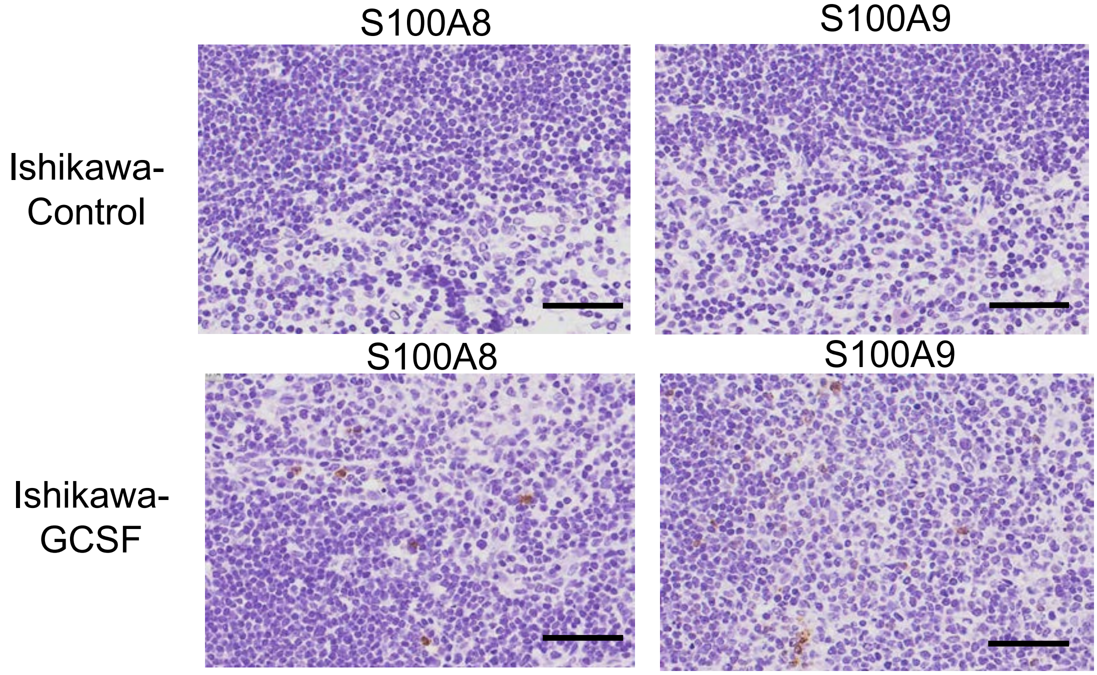


**Supplementary Fig. 1 Effects of tumor-derived G-CSF on the PALN and 18F-FDG-PET/CT in animal models of endometrial cancer. a** Representative image of G-CSF mRNA expression in Ishikawa-Control or Ishikawa-GCSF cells as evaluated by RT-PCR. **b** Representative G-CSF staining in Ishikawa-Control cells or Ishikawa-GCSF cells-derived tumors. **c** WBC/granulocyte counts of Ishikawa-Control-derived tumor- and Ishikawa-G-CSF-derived tumor-bearing rats (three rats per group). Rats were subcutaneously inoculated with Ishikawa-Control or Ishikawa-GCSF cells. Three weeks after inoculation, their subcutaneous tumors or peripheral blood samples were collected for analyses. **d** 18F-FDG-PET/CT scan of Ishikawa-Control-derived tumor-bearing rats and Ishikawa-GCSF-derived tumor-bearing rats. Rats were subcutaneously inoculated with Ishikawa-Control or Ishikawa-GCSF cells. Three weeks after inoculation, 18F-FDG-PET/CT was performed. Ishikawa-GCSF-derived tumor-bearing rats showed significant 18F-FDG-uptake in the PALN (circle). **e** PALNs resected after 18F-FDG-PET/CT. **f** Representative pathological findings from the PALNs resected after 18F-FDG-PET/CT (hematoxylin and eosin). Both of images contain no malignant cells. **g** Effects of tumor-derived G-CSF on the induction of MDSC in rat models of endometrial cancer. (i) and (ii), $CD11b/c^{+}HIS48^{+}$ cell populations detected in the peripheral blood and lymph nodes. Rats were subcutaneously inoculated with Ishikawa-Control or Ishikawa-GCSF cells. Three weeks after inoculation, the number of MDSC was evaluated by flow cytometry (three rats per group). **h** Representative immunoreactivities of resected paraaortic lymph nodes for S100SA8 and S100A9 (Ishikawa-Control-derived tumor- or Ishikawa-GCSF-derived tumor-bearing rats). Error bars indicate mean $\pm$ SD. Statistical significance was assessed using two-sided Welch *t* test. bp, base pairs. Scale bar, 50 μm. Source data are provided as a Source Data file.

Supplementary Fig. 2

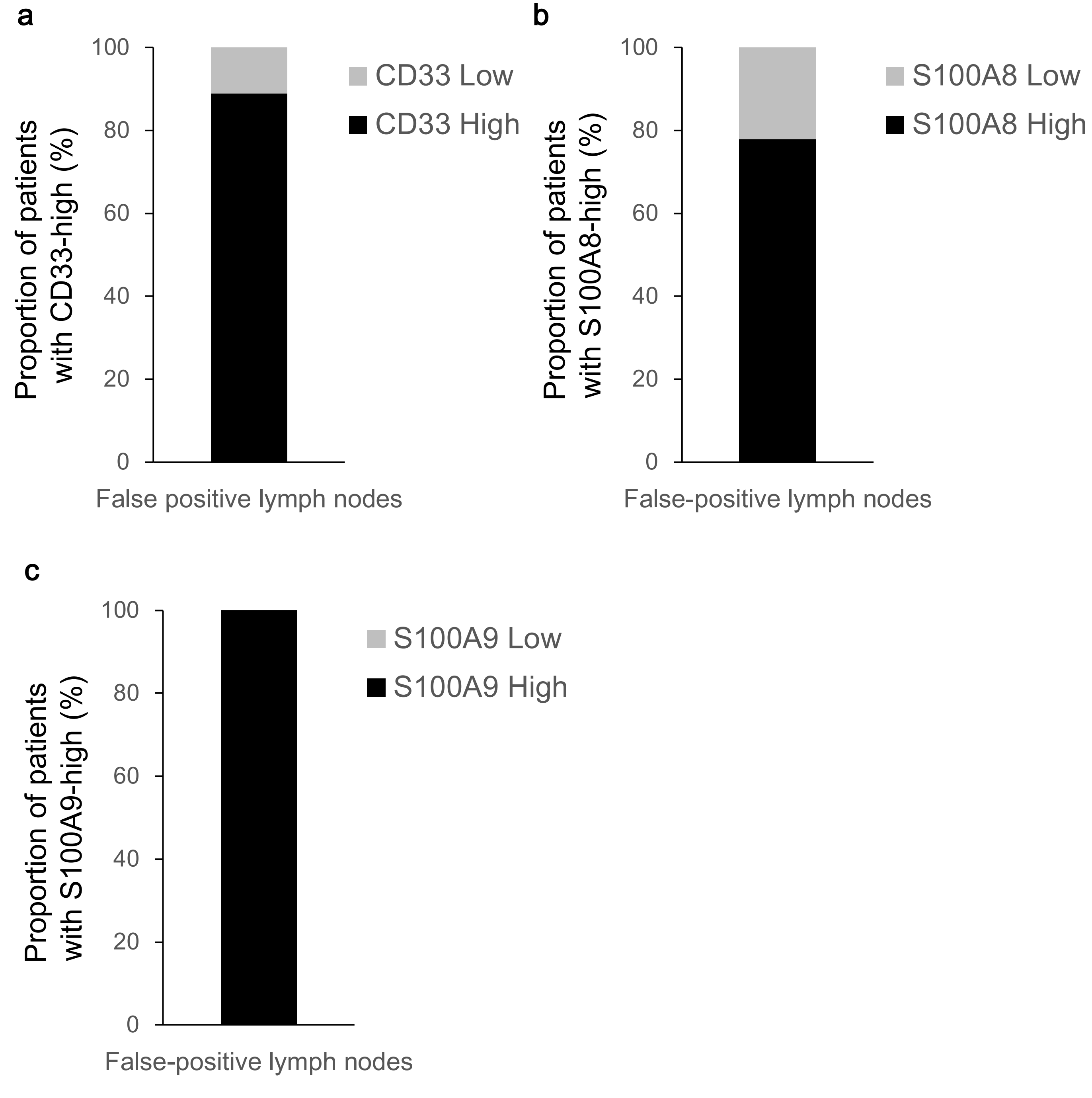


**Supplementary Fig. 2 Immunoreactivity of false-positively detected lymph nodes for CD33 and S100A8/9 (cervical cancer cases).** Of the 40 lymph nodes examined in Fig. 4A, nine were false-positively detected by 18F-FDG-PET/CT. Using these nice lymph nodes, immunohistochemical analyses were performed. **a** immunoreactivity of false-positively detected lymph nodes for CD33. **b** immunoreactivity of false-positively detected lymph nodes for S100A8. **c** immunoreactivity of false-positively detected lymph nodes for S100A9. Source data are provided as a Source Data file.

Supplementary Fig. 3

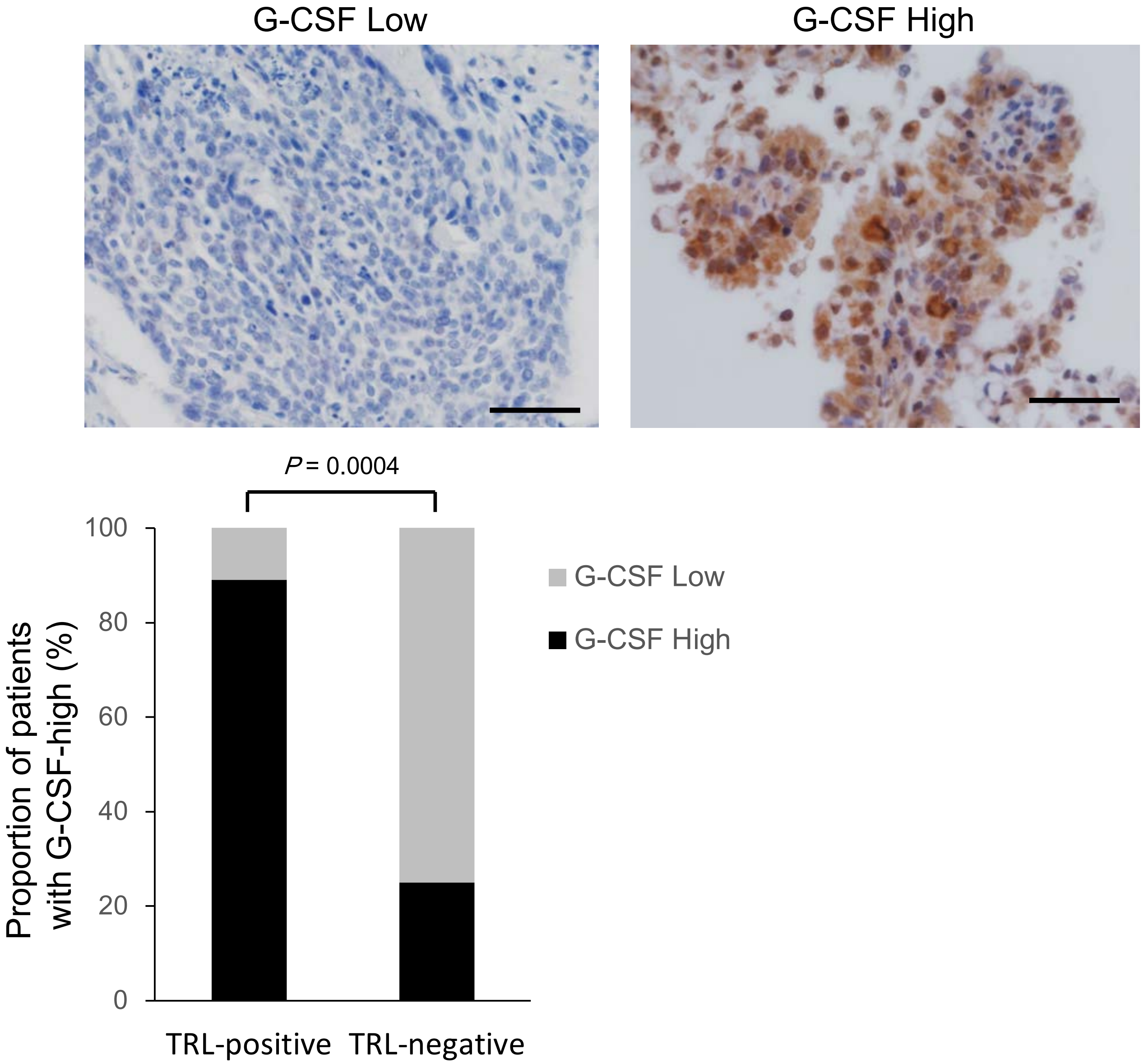


**Supplementary Fig. 3 G-CSF expression of the primary cervical tumor in patients with false-positive 18F-FDG-PET/CT results in lymph nodes (TRL-positive versus TRL-negative patients)**. Photographs; representative G-CSF-stained primary tumor sections of cervical cancer patients. A graph; proportion of patients with G-CSF-high. Scale bars, 50 $\mu$m. Statistical significance was assessed using two-sided Fisher's exact test (TRL-positive: WBC$\geq$ 9,000/mL; TRL-negative: WBC< 9,000/mL). Source data are provided as a Source Data file.

Supplementary Fig. 4

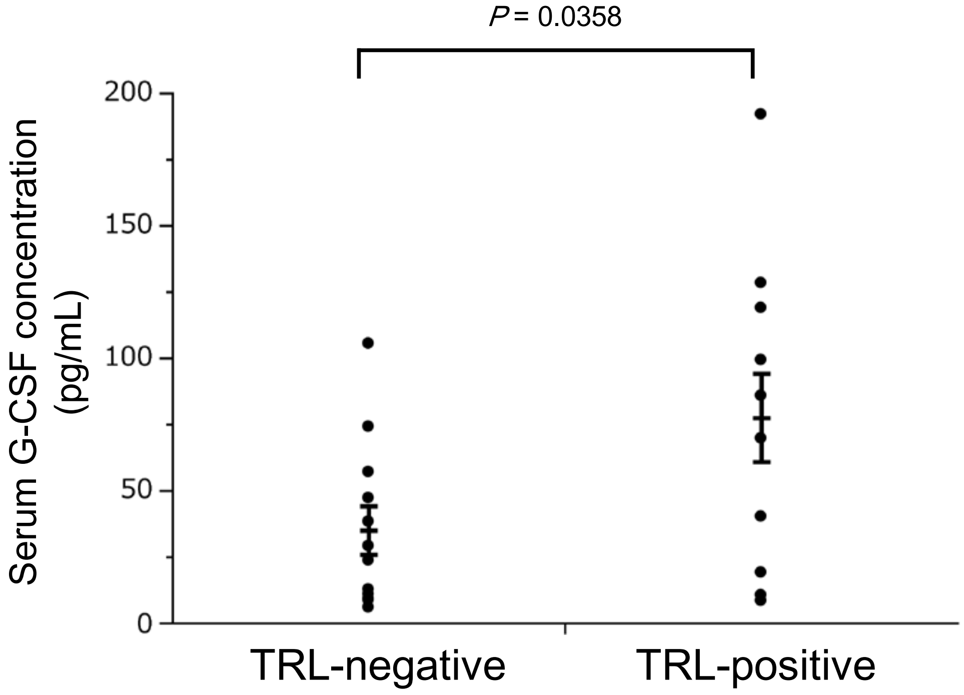


**Supplementary Fig. 4 The serum G-CSF concentrations in gynecological cancer patients included in the validation cohort [TRL-negative (n=12) versus TRL-positive (n=12)].** Error bars indicate mean ± SD. Statistical significance was assessed using two-sided Welch *t* test. Source data are provided as a Source Data file

Supplementary Fig. 5

**a** Cervical cancer

PFS (TRL-positive patients)

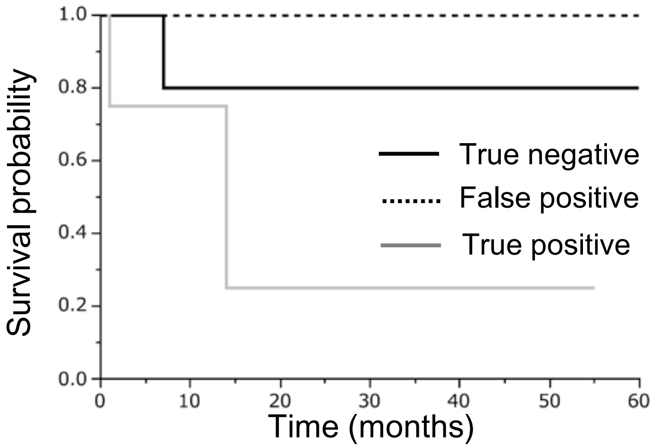


True negative vs False positive; p=0.1757
False positive vs True positive; p=0.0050
True negatve vs True positive; p=0.0002

OS (TRL-positive patients)

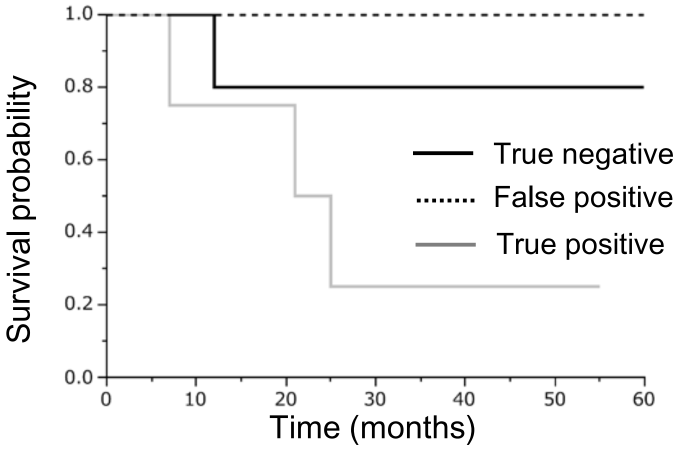


True negative vs False positive; p=0.3722
False positive vs True positive; p=0.0124
True negative vs True positive; p<0.0001

**b** Endometrial cancer

PFS (TRL-positive patients)

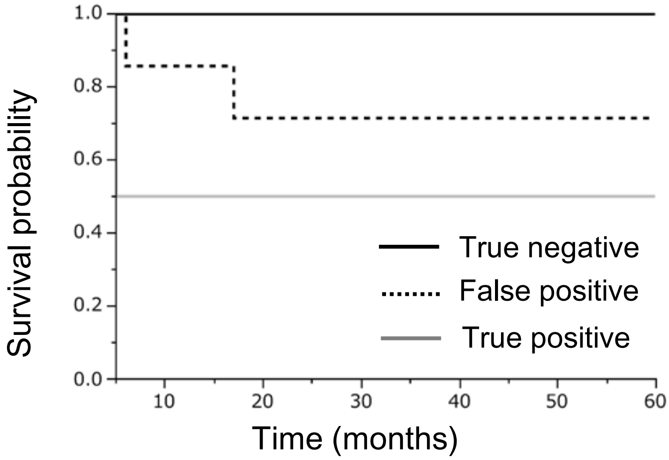


True negative vs False positive; p=0.2137
False positive vs True positive; p=0.4228
True negative vs True positive; p=0.1138

OS (TRL-positive patients)

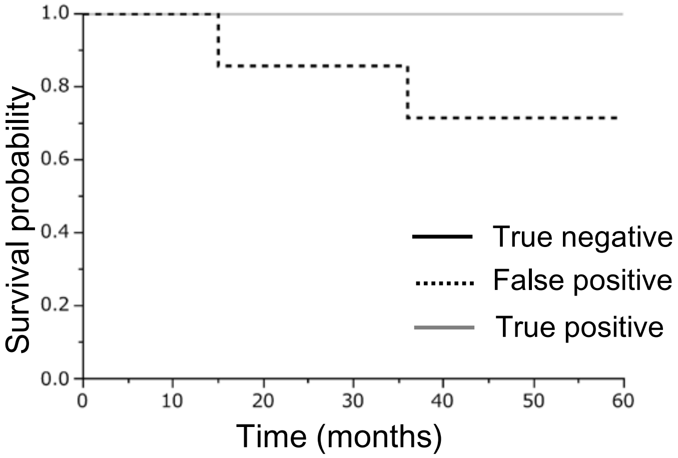


True negative vs False positive; p=0.2137
False positive vs True positive; p=0.4135
True negative vs True positive; Not available

**c** Ovarian cancer

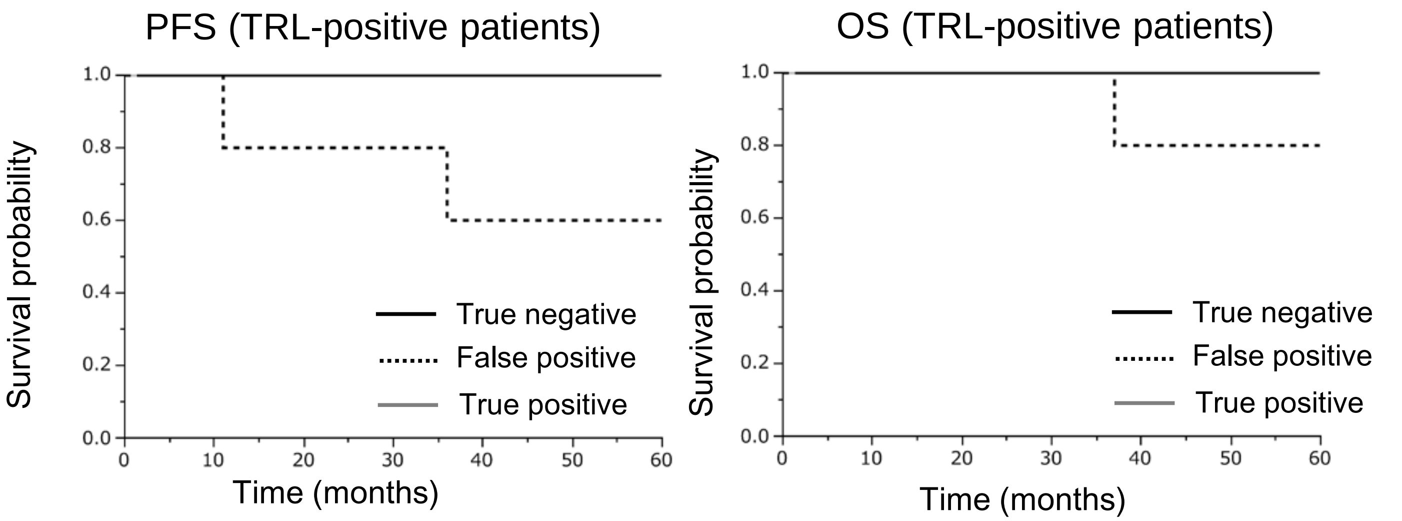


True negative vs False positive; p=0.1385
False positive vs True positive; Not available
True negative vs True positive; Not available

True negative vs False positive; p=0.3711
False positive vs True positive; Not available
True negative vs True positive; Not available

**Supplementary Fig. 5 Impact of lymph node statuses on progression free survival (PFS) and overall survival (OS) of TRL-positive gynecological cancer patients. a** Kaplan-Meier estimates of PFS and OS in TRL-positive cervical cancer patients. **b** Kaplan-Meier estimates of PFS and OS in TRL-positive endometrial cancer patients. **c** Kaplan-Meier estimates of PFS and OS in ovarian cancer patients. (True positive: 18F-FDG-PET/CT positive and pathologically positive; False positive: 18F-FDG-PET/CT positive and pathologically negative; True negative: 18F-FDG-PET/CT negative and pathologically negative; TRL-positive: WBC$\geq$ 9,000/mL). Statistical significance was assessed by log-rank test.

Supplementary Fig. 6

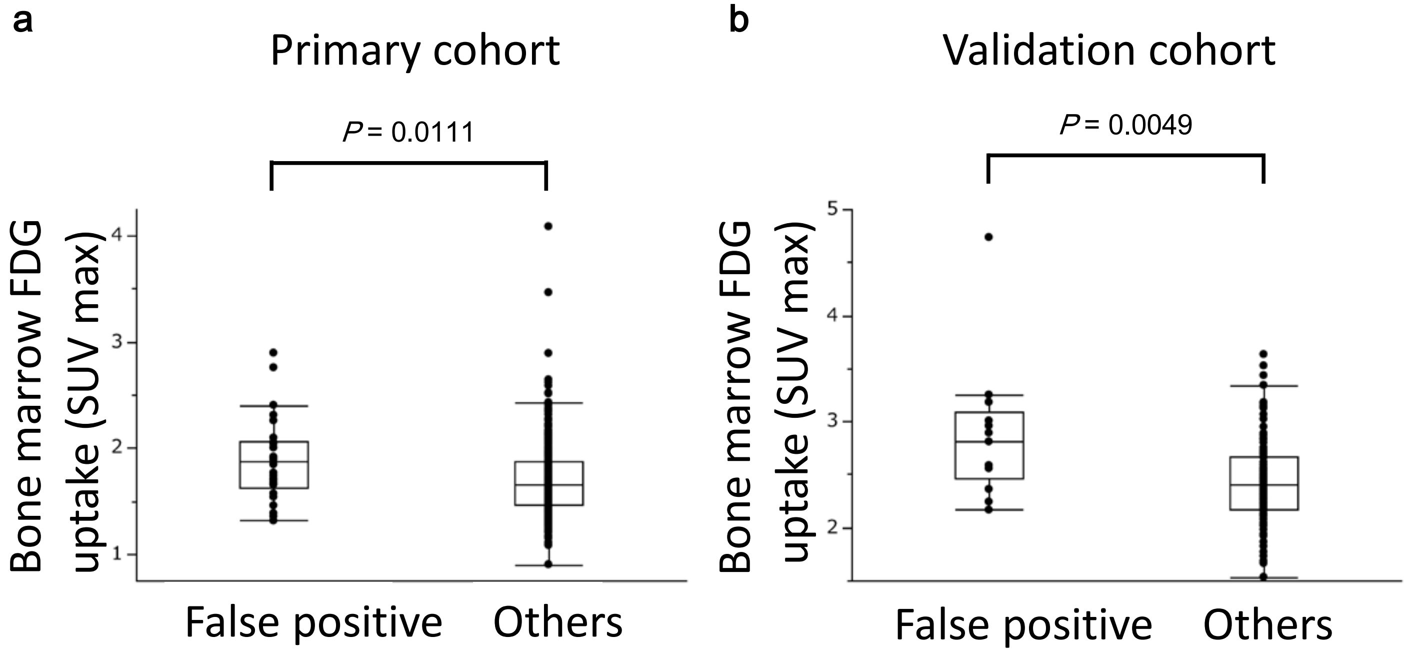


**Supplementary Fig. 6 Bone marrow FDG uptake in gynecological cancer patients (patients with false-positive 18F-FDG-PET/CT result versus those without). a** Primary cohort (n=424). **b** Validation cohort. Graphs are depicting the mean SUV max of bone marrow (thoracic vertebra 8-12) in each patient (n=118). Statistical significance was assessed using Wilcoxon rank-sum test. The box extends from 25th to 75th percentiles, the middle line denotes the median and the whiskers extend from the minimum to maximum value. Source data are provided as a Source Data file.

Supplementary Fig. 7

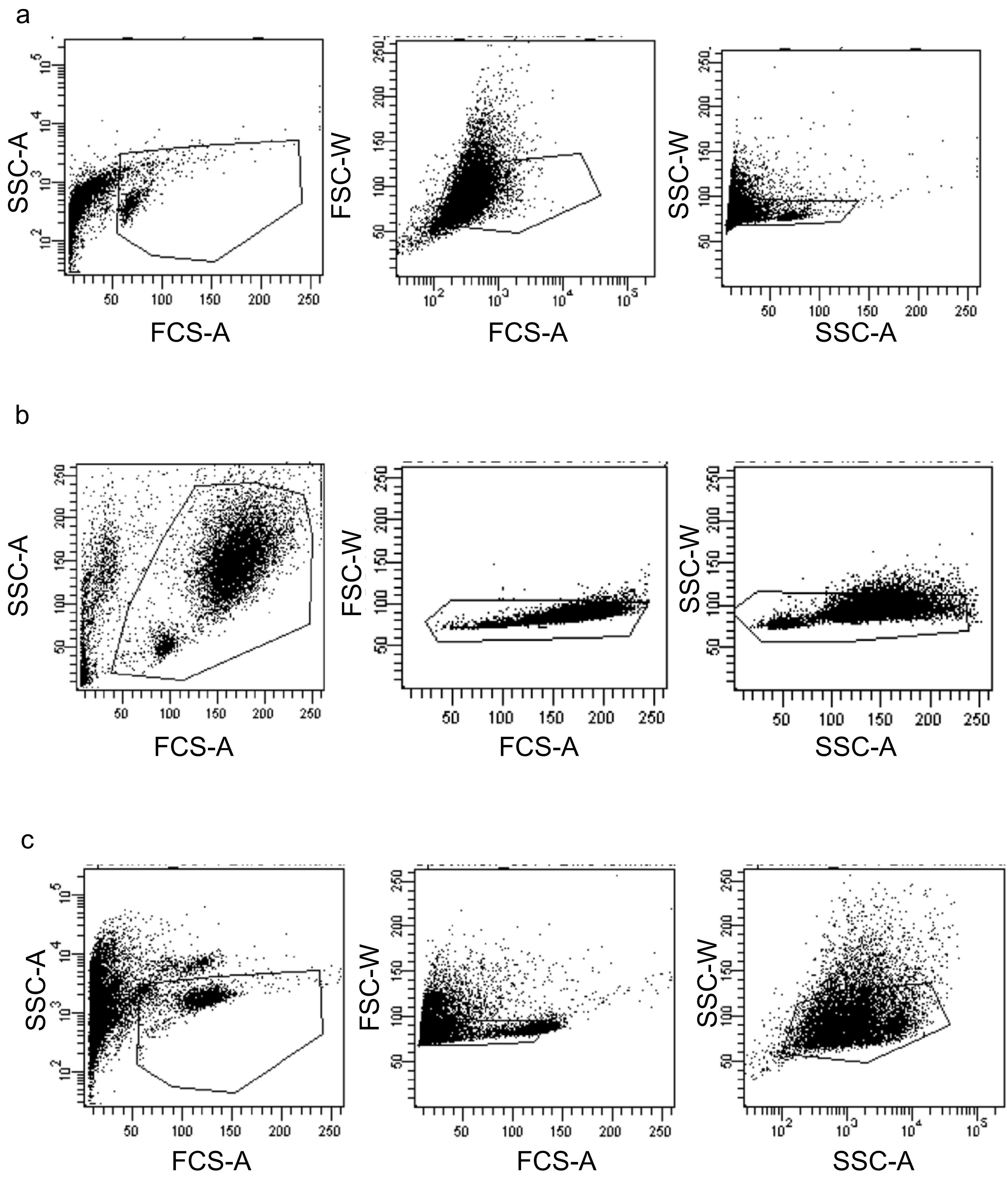


**Supplementary Fig. 7 Gating strategies for the flow cytometry. a** Gating strategies for Fig. 3g. **b** Gating strategies for Fig. 3i. **c** Gating strategies for Supplementary Fig. 1g.

Supplementary Table 1. 18F-FDG-PET/CT and histopathologic findings of lymph nodes during LN staging (primary cohort).

| | | Cervical cancer (n=127) | | Endometrial cancer (n=203) | | Ovarian caner (n=96) | |
|---|---|---|---|---|---|---|---|
| | | Histopathologic findings | | Histopathologic findings | | Histopathologic findings | |
| | | Positive (n=32) | Negative (n=95) | Positive (n=33) | Negative (n=170) | Positive (n=25) | Negative (n=71) |
| FDG-PET/CT findings | Positive | 13 (10.2) | 10 (7.9) | 15 (7.4) | 12 (5.9) | 14 (14.6) | 8 (8.3) |
| | Negative | 19 (15.0) | 85 (66.9) | 18 (8.9) | 158 (77.8) | 11 (11.5) | 63 (65.6) |

FDG, fluorodeoxyglucose; PET, positron emission tomography; CT, computed tomography

Supplementary Table 2. Clinicopathological characteristics of the patients with cervical, endometrial and ovarian cancer in validation cohort.

| | | All patients (n=125) | Cervical cancer (n=35) | Endometrial cancer (n=55) | Ovarian cancer (n=35) |
|---|---|---|---|---|---|
| | | n (%) | n (%) | n (%) | n (%) |
| Age (years) | <50 | 45 (36.0) | 22 (62.9) | 10 (18.2) | 13 (37.1) |
| | ≥51 | 80 (64.0) | 13 (37.1) | 45 (81.8) | 22 (62.9) |
| FIGO sage | I | 80 (64.0) | 26 (74.3) | 40 (72.7) | 14 (40.0) |
| | II | 20 (16.0) | 9 (25.7) | 4 (7.3) | 8 (22.9) |
| | III | 23 (18.4) | 0 | 10 (18.2) | 13 (37.1) |
| | IV | 2 (1.6) | 0 | 1 (1.8) | 0 |
| Histology | SCC | | 20 (57.1) | | |
| | Others | | 15 (42.9) | | |
| | EAC | | | 48 (87.3) | |
| | Others | | | 7 (12.7) | |
| | SAC | | | | 18 (51.4) |
| | Others | | | | 17 (48.6) |
| WBC count (/μL) | ≥9000 | 17 (13.6) | 4 (11.4) | 9 (16.4) | 4 (11.4) |
| | < 9000 | 108 (86.4) | 31 (88.6) | 46 (83.6) | 31 (88.6) |

FIGO, International Federation of Gynecology and Obstetrics; SCC, squamous cell carcinoma; EAC, endometrioid adenocarcinoma; SAC, serous adenocarcinoma; WBC, white blood cell

Supplementary Table 3. 18F-FDG-PET/CT and histopathologic findings of lymph nodes during LN staging (validation cohort).

| | | Validation cohort (n=125) | |
|---|---|---|---|
| | | Histopathologic findings | |
| | | Positive | Negative |
| FDG-PET/CT findings | Positive | 13 (10.4) | 13 (10.4) |
| | Negative | 23 (18.4) | 76 (60.8) |

FDG, fluorodeoxyglucose; PET, positron emission tomography; CT, computed tomography

Supplementary Table 4. The impact of pretreatment TRL on the diagnostic performance of 18F-FDG-PET/CT during LN staging in gynecological cancer patients (validation cohort).

| | | Validation cohort (n=125) | | |
|---|---|---|---|---|
| | | False-positive PET/CT results | | |
| | | Yes (n=13) | No (n=112) | P-value |
| WBC count (/μL) | <9000 | 7 (6.5) | 101 (93.5) | 0.0003 |
| | ≥9000 | 6 (35.3) | 11 (64.7) | |

FDG, fluorodeoxyglucose; PET, positron emission tomography; CT, computed tomography; WBC, white blood cell

Statistical significance was assessed using two-sided Fisher's exact test.
