# Supplementary material for: Pretreatment tumor-related leukocytosis misleads positron emission tomography-computed tomography during lymph node staging in gynecological malignancies
Source: Nat Commun. 2020 Mar 13;11:1364. doi: 10.1038/s41467-020-15186-z (PMC7069975; doi:10.1038/s41467-020-15186-z)
Supplement: Supplementary file 3 — Reporting Summary [file 41467_2020_15186_MOESM3_ESM.pdf]

nature research

Corresponding author(s): Seiji Mabuchi

Last updated by author(s): Jan 26, 2020

Reporting Summary

Nature Research wishes to improve the reproducibility of the work that we publish. This form provides structure for consistency and transparency in reporting. For further information on Nature Research policies, see Authors & Referees and the Editorial Policy Checklist .

Statistics

For all statistical analyses, confirm that the following items are present in the figure legend, table legend, main text, or Methods section.

n/a	Confirmed	
☐	☒	The exact sample size (*n*) for each experimental group/condition, given as a discrete number and unit of measurement
☐	☒	A statement on whether measurements were taken from distinct samples or whether the same sample was measured repeatedly
☐	☒	The statistical test(s) used AND whether they are one- or two-sided<br>*Only common tests should be described solely by name; describe more complex techniques in the Methods section.*
☐	☒	A description of all covariates tested
☐	☒	A description of any assumptions or corrections, such as tests of normality and adjustment for multiple comparisons
☐	☒	A full description of the statistical parameters including central tendency (e.g. means) or other basic estimates (e.g. regression coefficient) AND variation (e.g. standard deviation) or associated estimates of uncertainty (e.g. confidence intervals)
☐	☒	For null hypothesis testing, the test statistic (e.g. *F*, *t*, *r*) with confidence intervals, effect sizes, degrees of freedom and *P* value noted<br>*Give P values as exact values whenever suitable.*
☒	☐	For Bayesian analysis, information on the choice of priors and Markov chain Monte Carlo settings
☒	☐	For hierarchical and complex designs, identification of the appropriate level for tests and full reporting of outcomes
☒	☐	Estimates of effect sizes (e.g. Cohen's *d*, Pearson's *r*), indicating how they were calculated

Our web collection on statistics for biologists contains articles on many of the points above.

Software and code

Policy information about availability of computer code

Data collection	PET/CT examinations for human: Gemini GXL 16 (Philips) or SET-3000 GCT/X (Shimadzu)<br>PET/CT measurements for animals: Small-animal PET-CT scanner (Inveon MM; Siemens Medical Solutions)<br>Imuunohistochemistry images: PROVIS AX80 (Olympus)<br>Flow cytometry: BD FACSDiva software (version 6.1.1)
Data analysis	PET/CT images for human: Advantage Windows Workstation (version 4.5)<br>PET/CT measurements for animals: RadiAnt DICOM Viewer (version 4.6.9)<br>Statistical analysis: JMP® software (version 14.0) and GraphPad Prism (version 8.00)

For manuscripts utilizing custom algorithms or software that are central to the research but not yet described in published literature, software must be made available to editors/reviewers. We strongly encourage code deposition in a community repository (e.g. GitHub). See the Nature Research guidelines for submitting code & software for further information.

Data

Policy information about availability of data

All manuscripts must include a data availability statement. This statement should provide the following information, where applicable:

- Accession codes, unique identifiers, or web links for publicly available datasets
- A list of figures that have associated raw data
- A description of any restrictions on data availability

The source data underlying Fig. 3a, 3c, 3g, 3i, 3i, 4a, 4b and 5, and Supplementary Fig. 1a, 1c, 1g, 2, 3,4 and 6 are provided as a Source Data file. All other data that support the findings of this study are available within the article and its supplementary information files and from the corresponding author upon reasonable request.

Field-specific reporting

Please select the one below that is the best fit for your research. If you are not sure, read the appropriate sections before making your selection.

☒ Life sciences ☐ Behavioural & social sciences ☐ Ecological, evolutionary & environmental sciences

For a reference copy of the document with all sections, see nature.com/documents/nr-reporting-summary-flat.pdf

Life sciences study design

All studies must disclose on these points even when the disclosure is negative.

Sample size	A total of 551 gynecological cancer patients who had undergone a preoperative 18F-FDG-PET/CT scan before initial staging surgery at Osaka University Hospital were included in the current study. Because the samples were chronologically divided into two cohorts (the primary cohort (n=426); treated between April 2007 and December 2014, the validation cohort (n=125); treated from January 2015 to October 2018), the sample size for each cohort has not been determined.
Data exclusions	In human clinical data analyses, patients with coexisting hematologic malignancies, administration of corticosteroids or recombinant G-CSF, or acute or chronic infection were excluded, as these factors can be a cause of leukocytosis or a false-positive detection of lymph node metastasis by 18F-FDG-PET/CT.
Replication	All findings were reproducible, as more than 1 biological replicate was generally carried out, or the sample sizes were large enough to ensure the representation of the population behavior.
Randomization	Samples were not randomly allocated into experimental groups. Co-variates were controlled by performing isogenic comparisons.
Blinding	In vitro and in vivo studies, blinding was not relevant as samples were not randomly allocated to subgroup for analysis.<br>In human clinical data analyses, the identity of the patients was blinded by anonymisation of the samples. During assessment of quantification of IHC stainings, observers are blinded to the patient's clinical data.

Reporting for specific materials, systems and methods

We require information from authors about some types of materials, experimental systems and methods used in many studies. Here, indicate whether each material, system or method listed is relevant to your study. If you are not sure if a list item applies to your research, read the appropriate section before selecting a response.

Materials & experimental systems

n/a	Involved in the study	
☐	☒	Antibodies
☐	☒	Eukaryotic cell lines
☒	☐	Palaeontology
☐	☒	Animals and other organisms
☐	☒	Human research participants
☒	☐	Clinical data

Methods

n/a	Involved in the study	
☒	☐	ChIP-seq
☐	☒	Flow cytometry
☒	☐	MRI-based neuroimaging

Antibodies

Antibodies used	Antibodies used for immunohistochemistry:<br>Goat polyclonal anti-human G-CSF antibody (Santa Cruz Biotechnology, Cat#SC-1318, 1:200 dilution)<br>Mouse monoclonal anti-human CD33 antibody (Clone PWS44, Leica Biosystems Inc, Cat#NCL-L-CD33, Lot#6060905, 1:100 dilution)<br>Rabbit monoclonal anti-human S100A8 antibody (Clone MRP8, Abcam, Cat#ab92331, Lot#GR196390-8, 1:8000 dilution)<br>Rabbit polyclonal anti-rat S100A8 antibody (BOSTER BIOLOGY, Cat#PB9742, Lot#0971512Da8042109, 1:1000 dilution),<br>Rabbit polyclonal anti-human/rat S100A9 antibody (NOVUS Biologicals, Cat#NB110-89726, Lot#B-2, 1:2000 dilution).<br>Antibodies used for flow cytometry:<br>PE-conjugated anti-rat CD11b/c antibody(Clone REA325, Miltenyi Biotec, Cat#130-105-316, Lot#5171018298),<br>FITC-conjugated anti-rat HIS48 antibody (Clone HIS48, Invitrogen, Cat#11-0570-82, Lot#4329938),<br>FITC-conjugated anti-mouse CD11b antibody (Clone M1/70, Tonbo Biosciences, Cat#35-0112, Lot#C0112052014354)<br>APC-conjugated anti-mouse Gr1 antibody (Clone RB6-8C5, Biolegend, Cat#108411, Lot#B26854)<br>Antibodies used for T cell proliferation assay:<br>Purified anti-mouse CD3e antibody (Clone 145-2C11, Tonbo Biosciences, Cat#70-0031, Lot#P0031060617704)<br>For animal experiments:<br>Anti-mouse Gr-1 antibody (Clone RB6-8C5, BioXCell, Cat#BE0075, Lot#682618O1)

Validation	Anti-human G-CSF antibody (Santa Cruz Biotechnology), Anti-human CD33 antibody (Leica Biosystems Inc), anti-mouse CD11b antibody (Tonbo Biosciences), anti-mouse CD3e antibody (Tonbo Biosciences), and anti-mouse Gr-1 antibody (BioXCell) have been previously validated by our work (Mabuchi S, JNCI 2014, Kawano M, Sci Rep 2015). Anti-human S100A8 antibody (Abcam), anti-rat S100A8 antibody (BOSTER BIOLOGY), anti-rat/human S100A9 antibody (NOVUS Biologicals), anti-rat CD11b/c antibody (Miltenyi Biotec), anti-rat HIS48 antibody(Invitrogen), and anti-mouse Gr1 antibody (Biolegend) was validated as indicated on the manufacturer's website.

Eukaryotic cell lines

Policy information about cell lines

Cell line source(s)	ME180 human cervical cancer cell line and Ishikawa human endometrial cancer cell line were perchased from the American Type Culture Collection.
Authentication	Cells were authenticated by short tandem repeat method.
Mycoplasma contamination	Cells were routinely screened as negative for mycoplasma species (EZ-PCR Mycoplasma Test Kit; Biological Industries; #20-700-20).
Commonly misidentified lines (See ICLAC register)	No commonly misidentified cell lines were used.

Animals and other organisms

Policy information about studies involving animals; ARRIVE guidelines recommended for reporting animal research

Laboratory animals	BALB/c nude mice, female, 5- to 6-week-old; F344/NJcl-rnu/rnu rats, female, 7- to 8- week-old. Animals were maintained under specific-pathogen-free condition and reared at a temperature of 23 ± 1.5°C and a humidity of 45 ± 15%.
Wild animals	The study did not involve wild animals.
Field-collected samples	The study did not involve samples collected from the field.
Ethics oversight	All procedures involving mice and rats, and their care were approved by the Institutional Animal Care and Use Committee of the Osaka University in accordance with the relevant institutional and NIH guidelines.

Note that full information on the approval of the study protocol must also be provided in the manuscript.

Human research participants

Policy information about studies involving human research participants

Population characteristics	A total of 551 Japanese women with gynecological cancer who underwent pretreatment 18F-FDG-PET/CT scans were included (Detailed clinicopathological characteristics were described in Table 1 and Supplementary Table 2.
Recruitment	Patients with newly diagnosed gynecological cancers who had undergone a preoperative 18F-FDG-PET/CT scan before initial staging surgery at Osaka University Hospital (primary cohort, from April 2007 to December 2014; validation cohort, from January 2015 to October 2018) are included in the current study. We cannot exclude potential sources of bias: self-selection bias might have been introduced by the physicians when they perform 18F-FDG-PET/CT scan. As patients who had not undergone 18F-FDG-PET/CT scan were not included in this study, this might have led to over- or under- estimation of the results.
Ethics oversight	Permission to proceed with data acquisition and analysis was obtained from the institutional review board of Osaka University Hospital.

Note that full information on the approval of the study protocol must also be provided in the manuscript.

Flow Cytometry

Plots

Confirm that:

- [x] The axis labels state the marker and fluorochrome used (e.g. CD4-FITC).
- [x] The axis scales are clearly visible. Include numbers along axes only for bottom left plot of group (a 'group' is an analysis of identical markers).
- [x] All plots are contour plots with outliers or pseudocolor plots.
- [x] A numerical value for number of cells or percentage (with statistics) is provided.

Methodology

Sample preparation	Single cell suspensions were prepared from rat blood and lymph nodes. RBCs were removed using ammonium chloride lysis buffer. Cells were filtered through 40-µm nylon strainers, incubated with the appropriate antibody, and analyzed by flow cytometry.
Instrument	FACSCantoII flow cytometer (BD Bioscience)
Software	Data collection and analysis was performed using FACSDiva software (version 6.1.1).
Cell population abundance	No cell sorting was performed in the current study.
Gating strategy	Cells were identified using Forward and Side scatter and data from 10,000 cells sample was collected. Gating was used to measure the % CD11b/c and % HIS48 positive cells.

☒ Tick this box to confirm that a figure exemplifying the gating strategy is provided in the Supplementary Information.
